# Pulmonary artery sensor system pressure monitoring to improve heart failure outcomes (PASSPORT-HF): rationale and design of the PASSPORT-HF multicenter randomized clinical trial

**DOI:** 10.1007/s00392-022-01987-3

**Published:** 2022-03-04

**Authors:** Stefan Störk, Alexandra Bernhardt, Michael Böhm, Johannes Brachmann, Nikolaos Dagres, Stefan Frantz, Gerd Hindricks, Friedrich Köhler, Uwe Zeymer, Stephan Rosenkranz, Christiane Angermann, Birgit Aßmus

**Affiliations:** 1 Comprehensive Heart Failure Center, University and University Hospital Würzburg, Am Schwarzenberg 15, 97078 Würzburg, Germany; 2 Department of Internal Medicine I, University Hospital Würzburg, Würzburg, Germany; 3 Institut für Herzinfarktforschung (IHF GmbH), Ludwigshafen, Germany; 4 Department of Internal Medicine III, University Hospital, Saarland University, Homburg/Saar, Germany; 5 Department of Internal Medicine II, Klinikum Coburg GmbH, Coburg, Germany; 6 Leipzig Heart Institute, Leipzig, Germany; 7 Department of Electrophysiology, Heart Center Leipzig at the University of Leipzig, Leipzig, Germany; 8 Center for Cardiovascular Telemedicine, Charité Universitätsmedizin Berlin, Campus Mitte, Berlin, Germany; 9 Ludwigshafen Hospital, Ludwigshafen, Germany; 10 Department of Cardiology, Heart Centre Ludwigshafen, Ludwigshafen, Germany; 11 Department of Internal Medicine I, University Hospital Gießen and Marburg, Gießen, Germany

**Keywords:** Heart failure, Pulmonary artery pressure, Remote monitoring, CardioMEMS™ HF-System, Randomized controlled trial

## Abstract

**Background:**

Remote monitoring of patients with New York Heart Association (NYHA) functional class III heart failure (HF) using daily transmission of pulmonary artery (PA) pressure values has shown a reduction in HF-related hospitalizations and improved quality of life in patients.

**Objectives:**

PASSPORT-HF is a prospective, randomized, open, multicenter trial evaluating the effects of a hemodynamic-guided, HF nurse-led care approach using the CardioMEMS™ HF-System on clinical end points.

**Methods and results:**

The PASSPORT-HF trial has been commissioned by the German Federal Joint Committee (G-BA) to ascertain the efficacy of PA pressure-guided remote care in the German health-care system. PASSPORT-HF includes adult HF patients in NYHA functional class III, who experienced an HF-related hospitalization within the last 12 months. Patients with reduced ejection fraction must be on stable guideline-directed pharmacotherapy. Patients will be randomized centrally 1:1 to implantation of a CardioMEMS™ sensor or control. All patients will receive post-discharge support facilitated by trained HF nurses providing structured telephone-based care. The trial will enroll 554 patients at about 50 study sites. The primary end point is a composite of the number of unplanned HF-related rehospitalizations or all-cause death after 12 months of follow-up, and all events will be adjudicated centrally. Secondary end points include device/system-related complications, components of the primary end point, days alive and out of hospital, disease-specific and generic health-related quality of life including their sub-scales, and laboratory parameters of organ damage and disease progression.

**Conclusions:**

PASSPORT-HF will define the efficacy of implementing hemodynamic monitoring as a novel disease management tool in routine outpatient care.

**Trial registration:**

ClinicalTrials.gov; NCT04398654, 13-MAY-2020.

**Graphical abstract:**

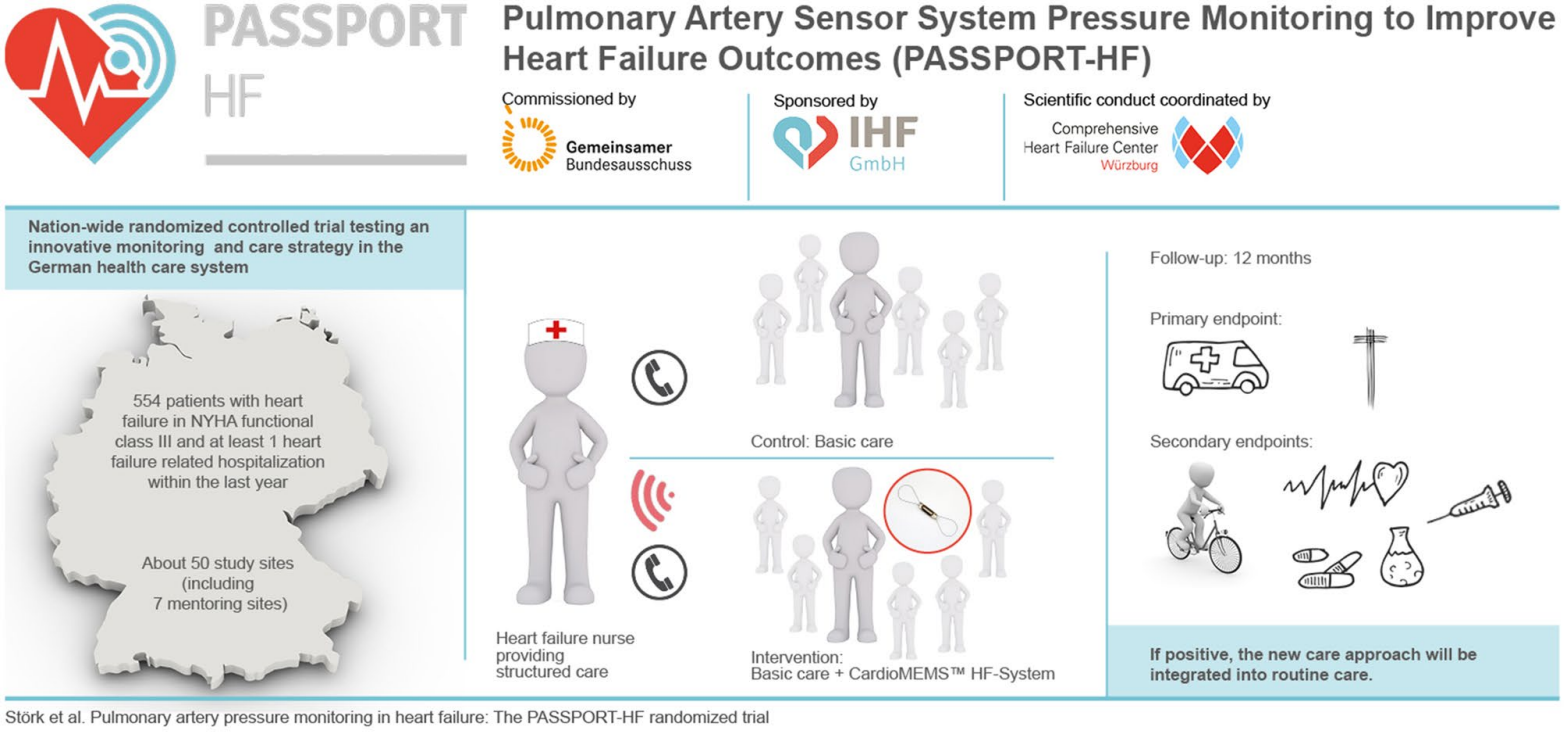

**Supplementary Information:**

The online version contains supplementary material available at 10.1007/s00392-022-01987-3.

## Introduction

Heart failure (HF) is a grave clinical syndrome characterized by complex treatment regimens, frequent rehospitalizations, and impaired quality of life [1]. For Germany, the annual incidence of HF was 655 per 100,000 persons estimated from health-care claims data sets [2], with an overall prevalence of 5.3% among adults [3]. Decompensation of HF in Germany accounts for more than 1,000,000 hospital admissions per year [2, 4], despite continuously improving treatment options [1]. Better implementation of and adherence to guideline-directed pharmacotherapy are regarded pivotal factors mediating more favorable outcomes [1, 5–7]. More than 90% of patients hospitalized for HF present with cardiopulmonary congestion, which develops secondary to an increased pulmonary arterial (PA) pressure. As this signal (or a clinical surrogate) is difficult to detect in routine care, electrode- and battery-free sensors have been developed that can wirelessly transmit PA pressure values from the patient's home by remote access [8].

The multicenter CHAMPION trial investigated 550 patients in New York Heart Association (NYHA) class III, who had been hospitalized within the previous year due to HF [9]. All patients were managed by dedicated HF staff. Care in the intervention arm was enhanced by utilizing information on PA pressure derived daily from a CardioMEMS™ sensor and transmitted wirelessly to the HF staff. Compared to controls, patients managed by PA pressure-guided care experienced 33% fewer HF-related hospitalizations within 15 months of follow-up [9]. Further, PA pressures were reduced, the proportion of patients hospitalized for HF decreased, and quality of life improved [9]. These effects were replicated when the former control group was also switched to CardioMEMS™-based remote care (risk reduction 48%) [10]. The 2016 ESC Guidelines for the diagnosis and treatment of acute and chronic HF assigned a IIb recommendation to the deployment of a CardioMEMS™ HF-System in HF patients meeting the selection criteria of the CHAMPION trial [11]. The European non-randomized MEMS-HF follow-up study assessed the safety and feasibility of the CardioMEMS™ HF-System in 234 NYHA class III patients [12]. MEMS-HF found that care enriched by remotely obtained PA pressure values was associated with an 80% (69%) reduction in HF (all-cause) hospital readmissions [13].

Although the CardioMEMS™ HF-System has demonstrated its safety and efficacy, all randomized evidence was generated in the USA. The German health-care system assigns different responsibilities to various care sectors without obligatory provision of a synchronized care plan including documentation and structured communication between care providers. Further, dedicated non-physician HF staff, e.g., HF nurses supporting patients and relatives during their iterative contacts with inpatient and outpatient care, are not yet part of clinical routine. The PASSPORT-HF trial was therefore initiated to prospectively investigate the efficacy of 12 months of PA pressure-guided remote HF management in HF patients with NYHA class III symptoms on clinical end points, within the German health-care system.

## Patients and methods

### Legal framework

The PASSPORT-HF trial was commissioned by the German Federal Joint Committee (G-BA), i.e., the highest decision-making body representing physicians, dentists, hospitals and health insurance funds, as part of a conditional coverage program in Germany for health-care-related costs. Interventions yielding benefits in trials executed under the auspices of the G-BA are to be implemented and reimbursed as part of routine care. As such, the G-BA provided the framework for the trial, outlined the PASSPORT-HF study design, and defined the primary end point. The G-BA provided funding for study conduct and case payments. Further, the G-BA passed regulations ensuring cost coverage by health insurance companies for the CardioMEMS™ HF-System, the dedicated patient electronics unit, and the post-discharge care both in the intervention and control arm, for duration of the trial.

### Study design, eligibility criteria for study sites and patients, and main hypothesis

**Fig. 1:**
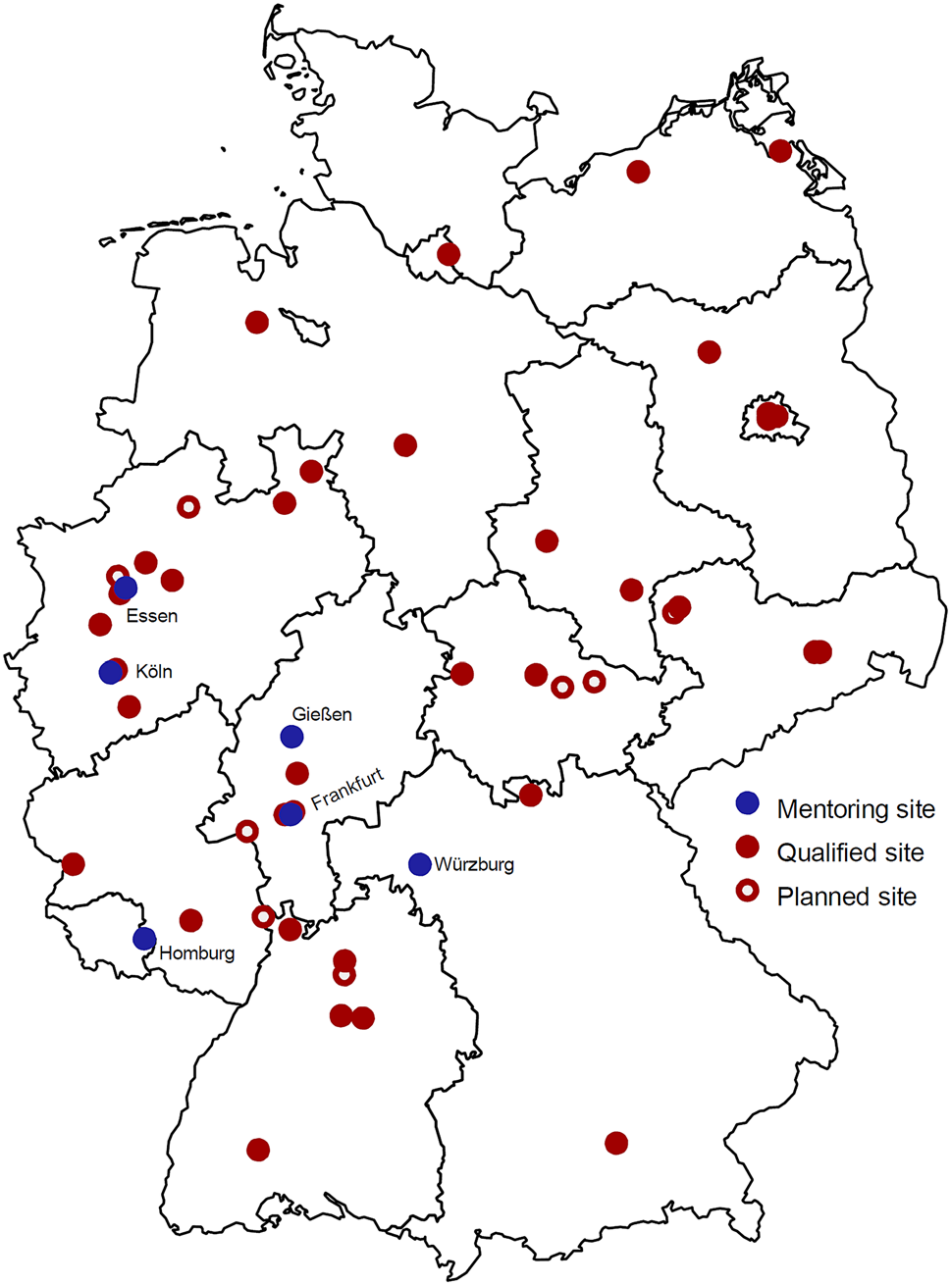
Participating centers in Germany

PASSPORT-HF is an open, prospective, randomized, multicenter clinical trial that will enroll 554 patients with chronic HF, predominantly in NYHA functional class III within the last 30 days and hospitalized for HF at least once in the 12 months prior to enrollment. Inclusion and exclusion criteria are shown in Tables 1 and 2. Diagnosis of HF is made by the study physician according to the criteria of the 2016 ESC Guidelines for Acute and Chronic Heart Failure [1, 11]. In total, about 50 German hospitals, cardiology practices, and medical service centers, distributed across all German federal states, will participate (Fig. 1; Supplemental Table S1). To qualify for an investigational site, specifically trained HF nurses or HF care experts must be available, and three CardioMEMS™ sensors must have been implanted and calibrated successfully. The primary hypothesis of the PASSPORT-HF trials states that the CardioMEMS™ HF-System applied in addition to basic HF care will reduce all-cause mortality and HF-related hospitalizations in patients with NYHA class III chronic HF.

**Table 1:** Inclusion criteria

1	Written informed consent received from the patient or a legal representative after the information has been provided
2	≥ 18 years of age
3	Predominant symptoms in NYHA Stage III in the 30-day period prior to consent to the study
4	Objectified HF diagnosis for more than 3 months
5	Hospitalization within 12 months prior to inclusion due to deterioration of HF symptoms
6	Able to tolerate dual antiplatelet therapy or anticoagulation therapy for 1 month after sensor implantation
7	Patients with reduced left ventricular ejection fraction (LVEF) ≤ 40% (assessed within 6 months prior to inclusion) must be treated with guideline-compliant HF pharmacotherapy; if one class of guideline-compliant medication is not tolerated, appropriate documentation must be supplied; patients must receive and tolerate at least one class of guideline-compliant medication; if no guideline-compliant medication is tolerated at all, the patient may not participate in the study
8	In patients with preserved LVEF (> 40%; assessed within 6 months prior to inclusion), comorbidities must be treated in accordance with guideline-compliant medication
9	Chest circumference (measured at axillary level) of less than 165 cm if BMI > 35 kg/m^2^
10	Willing and mentally and physically able to meet the requirements for follow-up and long-term basic care (this includes the long-term willingness of the patient, and of their relatives where relevant, to participate in PA pressure-based monitoring)
11	Appropriate domestic situation, defined as being accessible by telephone (via fixed or mobile network)^a^
12	For the intervention group: implantation is only performed if the diameter of the pulmonary artery branch intended for implantation is ≥ 7 mm (assessment will be made during the right heart catheterization)

**Table 2:** Exclusion criteria

1	Enrollment in another study with an active treatment arm
2	Severe cardiovascular event (e.g., myocardial infarction, open heart surgery, stroke, CRT implantation) in the 2 months prior to admission
3	Therapy-refractory heart failure in ACC/AHA stage D or new therapies that have taken place or are planned in the next 12 months (e.g., implantation of a left ventricular assist system / transplantation)
4	Active infection
5	History of recurrent (> 1 episode) pulmonary embolism and/or deep vein thrombosis
6	Continuous or intermittent chronic inotropic therapy
7	Estimated glomerular filtration rate (eGFR) < 25 ml/min
8	Life expectancy (according to the study physician's assessment) < 12 months
9	Severe, unrepaired congenital heart defect that would prevent implantation of the sensor
10	Severe valve vitium with planned intervention in the next 3 months
11	Presence of a mechanical right heart valve
12	Mental disorder that presumably (in the opinion of the study physician) has a negative impact on patient compliance or consent
13	Failure of the coordinating physician to approve if the patient is enrolled in an HF disease management program or comparable case management program^a^
14	Women of childbearing age with a positive pregnancy test at the time of inclusion

### Randomization

At the baseline visit, after signing the written informed consent, patients will be randomized centrally 1:1 into basic HF care plus CardioMEMS™ PA-guided remote care versus basic HF care alone. Randomization is done block-wise, stratified by study site. Crossover is allowed per clinical investigation plan and will not lead to termination of study participation. After randomization, the CardioMEMS™ sensor is required to be implanted within 14 days. A second informed consent form will be signed by patients in the intervention arm for the use of the Merlin.net™ website, where transmitted sensor values are being stored.

### CardioMEMS™ HF-System

**Fig. 2:**
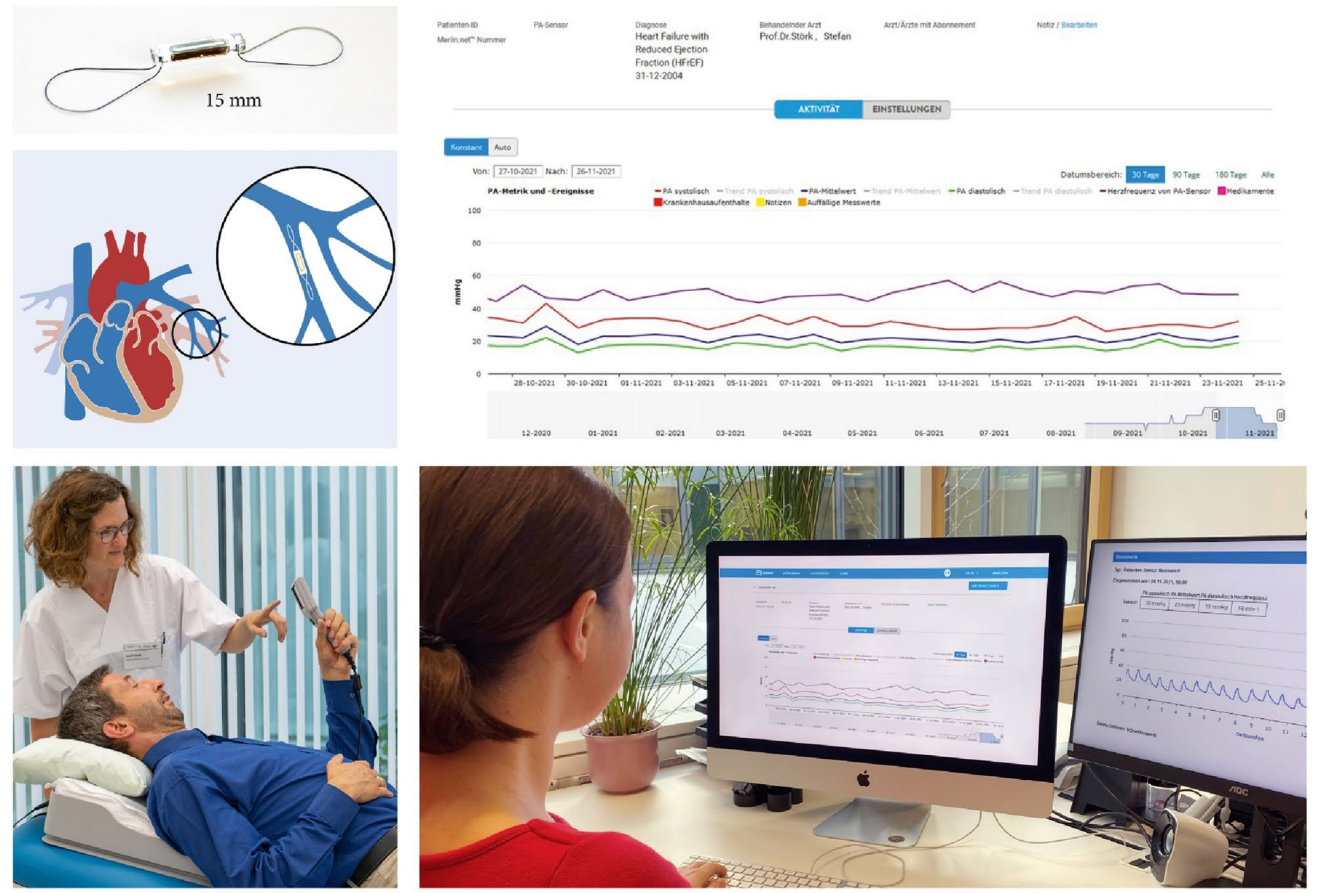
The CardioMEMS™ HF-System. The CardioMEMS pulmonary artery (PA) pressure sensor is shown (top, left), with its placement in the left PA. The sensor has a vertical orientation if the patient sits upright. After implantation, while still in hospital, the patient is instructed by trained staff how to position him/herself on the pillow that contains the measurement unit and measure and transmit the PA pressures once daily. The transmitted values can be accessed via a safe website by the heart failure nurse. Individual PA measurements (with tracings, see bottom right) and PA trends over time are visualized on the dashboard, allowing to interpret values in the context of supplementary information

All patients randomized into the intervention arm receive the CardioMEMS™ HF-System. It consists of three elements: (1) the PA pressure sensor (implanted into the distal branch of the descending pulmonary artery during right heart catheterization procedure via femoral vein approach), (2) the patient electronics system (a pillow placed in the patient's home environment including an electronic device with receiving and transmitting functions), and (3) the patient database (Merlin.net™ website), where the incoming PA pressure measurements are stored and visualized in trend graphics (Fig. 2) [9]. The device is CE marked for use in chronic HF patients in NYHA class III and with at least one HF hospitalization in the previous year [9]. Transmitted information consists of systolic, mean, and diastolic PA pressure trend information including the PA pressure waveforms in addition to pressure-derived heart rate. This information becomes instantly accessible and visible after measurement for review by clinicians and nurses via the Merlin.net website. A study-specific standard operating procedure has been produced serving as treatment guideline, particularly with regard to handling PA pressure values as possible triggers or supporting information for optimizing guideline-compliant HF treatment.

### Basic HF care

In the PASSPORT-HF trial, basic care is facilitated by HF nurses and comprises the five components summarized in Table 3.

**Table 3:** Definition of basic care in the PASSPORT-HF trial, delivered by specialized HF staff

Component	Description
Patient training at study start	An educational session with specialized HF staff while the patient is still in hospital; with the option to repeat educational segments during subsequent telephone calls, if required
Patient manual	An illustrated brochure handed out at study start; it informs on HF symptoms, medication, and gives advice regarding self-care, life-style adjustments, and emergency measures; during telephone follow-up calls, specialized HF staff can refer to its content
Symptom calendar	Booklet for daily self-recording of the patient´s body weight, blood pressure, heart rate, and symptoms
Repetitive structured telephone contact	Weekly calls in the first month after study initiation, then bi-weekly calls for patients in NYHA class III/IV, and 4-weekly calls for patients in NYHA class I/II for the remainder of the study period; contents of these contacts are documented in a structured fashion; a modified 14-item questionnaire [5] will be used addressing general health status, most recent self-measured values, indicators of deteriorating HF, well-being, and (changes in) medication; all educational elements relate to recommendations listed in the The German National Disease Management Guideline “Chronic Heart Failure” (*Nationale VersorgungsLeitlinie Chronische Herzinsuffizienz,* https://www.leitlinien.de/nvl/herzinsuffizienz)
Usual medical care	Treatment of HF and concomitant diseases by means of guideline-directed therapy and routine pathways established at the respective site or region

### CardioMEMS™ HF supported care

**Fig. 3:**
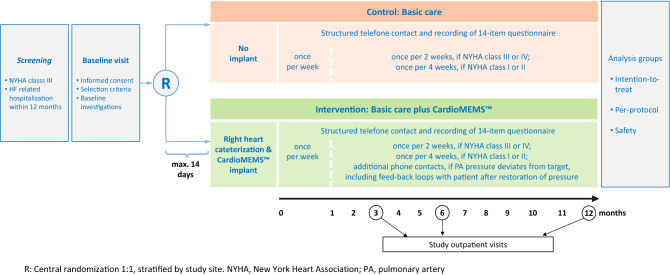
PASSPORT-HF trial follow-up scheme. R: central randomization 1:1, stratified by study site. NYHA, New York Heart Association; PA, pulmonary artery

In the intervention arm, the care concept includes all elements of basic care detailed in Table 3 and is further enhanced by using the PA pressure information transmitted daily via the CardioMEMS™ HF-System. After implantation and prior to hospital discharge, the HF nurse instructs the patient on the use of the measurement unit, frequency of measurements and transmissions, meaning of reaching optimal PA pressure targets and importance of euvolemia, intensified pharmacotherapeutic regimens to reach PA pressure targets, and the mode of telephone interaction and feedback loops that is now implemented for the study period (Fig. 3).

### Clinical study flow

In the light of the findings of the MEMS-HF study [12], we assumed an inclusion rate of one subject per month and site. With approximately 45 (40–50) enrolling sites, it is expected that the period of study enrollment will be around 18 months. Taking into account an interval for the initiation of the sites (approx. 6 months), this results in a planned enrollment phase of 24 months. All patients will be followed for 12 months. The follow-up visits are scheduled at 3, 6, and 12 months following randomization.

#### Patient visits

At the baseline visit, demographic data, medical history and current medication are recorded. Baseline assessment also includes a physical examination, NYHA functional class assessment, detailed laboratory evaluation, and questionnaires addressing quality of life and psycho-emotional domains (Kansas City Cardiomyopathy Questionnaire [KCCQ]; European Quality of Life 5 Dimensions 5-Level Version [EQ-5D-5L]; Patient Health Questionnaire Depressive Symptoms [PHQ-9]; Generalized Anxiety Disorder Scale [GAD-7]). For the intervention group, procedural details on the implantation procedure (including calibration of the PA sensor; adverse events and device/system-related complications) are documented. A standardized echocardiographic examination protocol is desirable, but for pragmatic reasons not obligatory. Investigations are repeated during outpatient visits after 3, 6, and 12 months (for details see Table S2).

#### Impact of the COVID-19 pandemic

Follow-up visits might require adjustment during the COVID-19 pandemic according to potential restrictions imposed by the government and consequences concerning the per-protocol conduct of the study. These may extend to travel restrictions, visiting bans regarding the investigational sites, or the study patient’s lacking willingness to visit the investigational site for the stipulated follow-up visits. If a study participant cannot or is unwilling to visit the investigational site in person, efforts will be taken to complete remotely (i.e., via telephone call) as much of the follow-up information as possible. Patient questionnaires will be sent to the patient by mail. Laboratory values substituting the per-protocol central laboratory can be obtained from the patient’s treating physician.

### Outcome measures

#### Primary end point

The primary efficacy end point is a composite of all-cause death and unplanned HF hospitalizations 365 days after randomization. Unplanned HF-related hospitalization is defined as an unplanned overnight hospitalization, the main reason for which is decompensated HF. Hospitalization due to HF also includes hospitalization resulting from the investigational intervention (e.g., bleeding). The primary safety end point is freedom from device/system-related complications (DSRC) in patients in whom an implantation has been tried or completed, or sensor failure, within a time frame of 12 months.

#### Secondary end points

The major secondary end point is the change in quality of life scores measured after 6 and 12 months using the Kansas City Cardiomyopathy Questionnaire (KCCQ). The KCCQ is a well-established measure for quality of life in HF patients and has been applied as end point in pharmacotherapeutic and device trials [14]. Additional secondary end points include general health-related quality of life (EQ-5D-5L at 6 and 12 months), mortality at 12 months (HF-related, cardiovascular, all cause), unscheduled hospitalizations (HF-related, cardiovascular, all cause, number of days alive and out of hospital), adverse events and symptoms of HF (Table 4).

**Table 4:** Study end points

Primary efficacy end point	Composite of the number of unplanned HF-related rehospitalizations or all-cause mortality 365 days after randomization (12-month time)
Primary safety end point	1. Device/system-related complications (DSRC) of the patients as a result of the attempted or successful implantation of an CardioMEMS™ sensor at the 12-month time point and 2. Freedom from sensor failures at the 12-month time
Secondary end points	(A) Health-related quality of life Major secondary: changes in quality of life (QoL) measured using the Kansas City Cardiomyopathy Questionnaire (KCCQ) scores (TSS, OSS, CSS), after 6 and 12 months Changes in QoL scores measured after 6 and 12 months using Euro-QoL-5D (B) Mortality HF-related mortality in the 12-month period Other cardiovascular mortality in the 12-month period Non-cardiovascular mortality in the 12-month period All-cause mortality in the 12-month period (C) Unplanned hospitalizations HF-related hospitalizations in the 12-month period Other cardiac-related hospitalizations in the 12-month period Non-cardiovascular-related hospitalizations in the 12-month period All-cause hospitalizations in the 12-month period Number of days alive and out of hospital in the 12-month period (D) Adverse events Frequency of adverse events in the 12-month period Frequency of serious adverse events in the 12-month period (E) Symptoms of heart failure and psychometric assessments Patient-reported symptoms of heart failure assessed by the KCCQ Symptoms Score Unscheduled HF-related hospitalizations HF-related mortality Days alive and out of hospital Laboratory parameters for organ damage and disease progression Change in symptom burden of anxiety (GAD-7) at 6- and 12-month time points Change in symptom burden of depressive symptoms (PHQ-9) at 6- and 12-month time points (F) Care-related aspects Patient adherence in the intervention group in terms of obtaining PA pressure readings, at 6 and 12 months Change in PA pressure values at 6 and 12 months, based on the area under the curve Number of adjustments made within each guideline-recommended substance class and their underlying reasons Change in drug dose (equivalent dosages) of maximum guideline-recommended substance class received, at 6 and 12 months Rate of documented atrial and ventricular arrhythmias over a 12-month period Laboratory measures of organ damage and disease progression: renal and cardiac biomarkers with associated changes Health economic data (resource use) at 6- and 12-month time points

### Sample size calculation

For PASSPORT-HF, we defined inclusion and exclusion criteria comparable to the CHAMPION trial and anticipated similar clinical effects [9, 10]. Accordingly, a rate of 0.84 events per patient-year was assumed for the primary efficacy end point in the control arm, and a rate of 0.58 events per patient-year in the intervention arm (corresponding to a hazard ratio of 0.69). Assuming a dropout rate of 20%, this yielded a total sample size of 554 patients for the study (227 patients per arm, assuming alpha = 2.5% for one-tailed testing, 1-beta = 90%). Accordingly, at least 442 patients, i.e., 221 patients per arm, are required for the proposed analysis using the Anderson–Gill model.

### Data analysis and analysis populations

#### Descriptive analyses

Data will be summarized using univariable statistics (number, mean, standard deviation, median, maximum, minimum) or frequency (absolute number, percentage). Between-group comparisons for baseline characteristics will be performed with Pearson’s Chi-squared test for categorical variables and Mann–Whitney or Wilcoxon tests for continuous variables.

#### Analysis populations

The primary hypothesis will be analyzed using the full analysis set based on the intention-to-treat (ITT) principle, i.e., on the data from all randomized patients (excluding only patients from the intervention/control group who terminate the study before the first implantation attempt; if the implantation is unsuccessful, the patient, however, will be included in the analysis set). A supportive per-protocol analysis of the primary hypothesis is performed (efficacy sample), including all who completed the study according to the clinical investigation plan and excluding patients violating the inclusion or exclusion criteria upon enrollment as well as patients with major deviations from the clinical investigation plan.

#### Primary end point analysis

The primary time point for the efficacy analysis on the reduction of unplanned HF hospitalizations or all-cause death is 12 months. Event rates will be compared between the intervention and control groups using a two-sided test with a significance level of 5%.

### Trial structure, registration and organization

The PASSPORT-HF trial is designed, implemented and supervised by the Steering Committee composed of major sites involved in the grant acquisition. The University Hospital Würzburg, German Center for Heart Failure (CHFC), is responsible for scientific coordinating management. The study and data management are performed by the CRO IHF GmbH, which also acts as sponsor. To ensure optimal speed of enrollment without compromising quality of care and documentation, so-called mentoring sites are established to accompany investigation sites at the start. Mentoring sites serve as a contact point for any content-related issues over the course of the study, supporting about seven to eight investigational sites during the first 6 months following the first randomization of an intervention patient at the investigational site. Mentoring activities include detailed instruction of the study team on coaching algorithms and PA pressure-guided options/requirements for therapeutic adjustment and telephone conferences on a regular basis (every 2 weeks) between the lead nurse of the mentoring site and study nurses of the respective investigation site to discuss any open issues and the implementation of the PA pressure-associated procedures on both the patient’s side as well as on the side of the study personnel. For further information, please refer to Supplemental Materials D.

### Re-imbursement strategies, trial funding, and role of device industry

Since the trial has been commissioned by the G-BA with the intention to implement PA pressure monitoring into routine care if the trial meets the primary end point, trial funding rests on two pillars. Study-related costs are compensated via the sponsor (G-BA). Further, all costs that potentially will become part of the future routine care are compensated by the respective general health insurance of a study participant. Therefore, a re-imbursement schedule has been regulated by the G-BA that anticipates the future routine costs including the hospitalization-related costs (3 days in hospital, right heart catheterization, PA pressure sensor, implantation procedure) and post-discharge-related costs (measurement unit, structured aftercare). The sensor manufacturer is not involved in any aspect of study design, conduct, data analysis, and reporting. The role of the sensor manufacturer has been refined to implantation training prior to initiation of a study site, technical assist during implantation, and solving technical queries related to the data transmission and the database (Merlin.net™).

## Discussion

The PASSPORT-HF trial has been designed to quantify the safety and the clinical benefits of PA pressure-guided HF care in ambulatory patients in the German health-care system. The study population is characterized by a marked, yet stabilized symptom burden (predominantly NYHA functional class III) regardless of the severity of left ventricular compromise. PASSPORT-HF is powered to determine whether PA pressure-guided remote monitoring will prevent episodes of decompensation and thereby translate into a reduction of unplanned HF-related rehospitalizations or death occurring up to 12 months after randomization.

Health-care providers and payers attribute increasing importance to preventive strategies to avoid worsening HF requiring hospital admission in outpatients. Various interventions have been tried to decrease event rates, including nurse-led disease management with patient self-monitoring of body weight and clinical features [5, 15], non-invasive telemedical systems [16], and remote HF management based on implantable electronic devices [17]. Meta-analyses suggest a heterogeneous impact on hard clinical and patient-reported end points, compatible with the view that not only the technical aspects (like type of sensor, mode of transmission), but also patient disposition, health-care setting, and the pattern, intensity and frequency of interaction between patient and carers determine the efficacy and effectiveness of remote HF care [18, 19].

Although the CardioMEMS™ HF-System has proven its safety, efficacy and sustainability of effects in the USA [9, 10], uptake of the system in the German health-care setting has been slow. This may be due to the fact that the PA pressure-guided remote monitoring approach relies on a structured care system for patients leaving the hospital after an episode of cardiac decompensation. Further, PA pressure monitoring is intersectoral by definition: it involves inpatient and outpatient caregivers and thus demands a dedicated communication and documentation system. To ensure optimal comparability, the G-BA relied on the CHAMPION trial and the non-randomized MEMS-HF as role models. It is the declared aim of PASSPORT-HF to create—via the conduct of the trial—the scientific basis for a comprehensive nurse-led post-discharge care structure based on PA pressure-guided remote monitoring. Because the contracting authority is the public health authority, PASSPORT-HF had to be as economical and as close to the envisioned future routine care as possible. Therefore, the trial is embedded into routine care and takes a pragmatic approach. Particular care was spent on a harmonized level of care and knowledge of all staff involved in patient selection, implantation, and aftercare. PA pressure-guided care is entrusted to dedicated staff, who successfully passed the HF nurse education curriculum. Further, to speed up the process of monitoring and coaching, so-called mentoring sites were installed as an additional feature. For a limited period of 6 months, mentoring sites assist newly beginning study sites and provide advice regarding all practical questions both with the study and the monitoring routine. Bi-weekly telephone conferences have been implemented as an obligatory feature to assist this process.

Consistent with results from the CHAMPION trial, the MEMS-HF prospective follow-up study also found that post-discharge care enhanced by remote PA pressure monitoring lowers PA pressure and favorably impacted on clinical end points and quality of life over a period of 12 months [12]. However, results from the recent GUIDE-HF trial, conducted in the USA and Canada, suggest that the success of PA pressure-guided care may vary across risk groups [20]. Successful implementation of remote care into clinical routine depends on several key factors that all must be installed and, ideally, quality checked over time. This includes (a) careful selection of a patient willing to adhere long-term to daily measurement and data transmission; (b) provision of expert staff (cardiologist, HF nurse) with dedicated time windows for iterative and structured contact with patient (and relatives), other caregivers (in particular general practitioners and co-treating cardiologist), and also the structured discussion within the telemedical care team itself; (c) care pathways with standard operating procedures that can be quality controlled and are properly reimbursed. Ideally, a telemedical center with an HF care network (e.g., [21]) should be present. The German health-care system currently accelerates the implementation of telecare options, also, in the context of HF [22]. These efforts are complemented by the implementation of a “disease management program heart failure” that aims to incentivize the adoption of a structured care plan for HF patients by general practitioners. This program will also allow for prescribing remote monitoring and managing components using novel sensors and approaches. Hence, the current PASSPORT-HF trial will help to pave the path for a balanced care depending on the severity of symptom burden and adopted to the needs of the patient.

### Strengths and limitations

One particular strength of PASSPORT-HF lies in the detailed a priori description of care strategies and patient interactions mandated by the study protocol. It is derived and adheres in large parts to the HeartNetCare-HF™ program employed in the randomized INH trial and later in the MEMS-HF study [5, 12], and is directed toward fostering patient self-care and empowerment through a combination of monitoring, education, and coaching. This system is highly structured, albeit flexible, and can easily incorporate additional information, e.g., remotely provided sensor signals. Owing to the pragmatic nature of the study design, certain diagnostic features are not obligatory, e.g., a high-end echocardiography scan that has been validated by central reading. The trial started recruiting in August 2020, but has been delayed by the COVID-19 pandemic. Particular precautions have been met to conduct the trial with similar rigor despite the challenges posed by the pandemic.

## Conclusions

PASSPORT-HF is a health authority-sponsored randomized controlled trial, which specifically has been designed to address the impact of hemodynamic-guided HF management in the German health-care setting, focusing on cumulative HF events and all-cause mortality. Additionally, the PASSPORT-HF trial will investigate the concomitant effects of hemodynamically guided remote monitoring on changes in disease-specific and general health-related quality of life and surrogates of organ damage.

## Supplementary Information

Below is the link to the electronic supplementary material.

Supplementary file1 (DOCX 36 kb)

## Data Availability

Not applicable.
