# Supplementary material for: Pulmonary artery sensor system pressure monitoring to improve heart failure outcomes (PASSPORT-HF): rationale and design of the PASSPORT-HF multicenter randomized clinical trial
Source: Clin Res Cardiol. 2022 Mar 4;111(11):1245–55. doi: 10.1007/s00392-022-01987-3 (PMC8896072; doi:10.1007/s00392-022-01987-3)
Supplement: Supplementary file 1 — Supplementary file1 (DOCX 36 kb) [file 392_2022_1987_MOESM1_ESM.docx]

**SUPPLEMENTARY MATERIALS**

**Pulmonary Artery Sensor System Pressure Monitoring to
Improve Heart Failure Outcomes (PASSPORT-HF):**

**Rationale and design of the PASSPORT-HF Multicenter Randomized Clinical Trial**

Stefan Störk^1,2^ ● Alexandra Bernhardt^3^ ● Michael Böhm^4^ ● Johannes Brachmann^5^
● Nikolaos Dagres^6,7^ ● Stefan Frantz^1,2^ ● Gerd Hindricks^6,7^ ● Friedrich Köhler^8^
● Uwe Zeymer^3,10^ ● Stephan Rosenkranz^9^ ● Christiane Angermann^1^ ● Birgit Aßmus^11^

***Affiliations***

1 Comprehensive Heart Failure Center, University and University Hospital Würzburg, Germany

2 Dept. of Internal Medicine I, University Hospital Würzburg, Germany

3 Institut für Herzinfarktforschung (IHF GmbH), Ludwigshafen, Germany

4 Dept. of Internal Medicine III, University Hospital, Saarland University, Homburg/Saar, Germany

5 Dept. of Internal Medicine II, Klinikum Coburg GmbH, Coburg, Germany

6 Leipzig Heart Institute, Leipzig, Germany

7 Dept. of Electrophysiology, Heart Center Leipzig at the University of Leipzig, Leipzig, Germany

8 Center for Cardiovascular Telemedicine, Charité Universitätsmedizin Berlin, Campus Mitte, Berlin, Germany

9 Ludwigshafen Hospital, Germany

10 Heart Centre Ludwigshafen, Department of Cardiology, Ludwigshafen, Germany

11 Dept. of Internal Medicine I, University Hospital Gießen and Marburg, Gießen, Germany

**CONTENT Page**

1. Table S1. List of participating study sites and principal investigators at study start ..........................2
2. Table S2. Time course of study procedures and patient contacts ……………………………………………….….4
3. Further statistical analyses ………………………………………………………………………………………………..………….5
4. Trial organization ................................................................................................................................6

# Table S1. List of participating study sites and principal investigators at study start

| **Location** | **Hospital of Medical Service Center** | **Local Principal Investigator** |
| --- | --- | --- |
| ***Mentoring Site*** | | |
| Essen | Elisabeth-Krankenhaus, Contilia Herz- und Gefäßzentrum | PD Dr. med. Oliver Bruder |
| Frankfurt | Universitätsklinikum Frankfurt | PD Dr. med. Mariuca Vasa-Nicotera |
| Gießen | Universitätsklinikum Gießen–Marburg GmbH | Prof. Dr. med. Birgit Aßmus |
| Homburg | Universitätsklinikum des Saarlandes | Prof. Dr. med. Michael Böhm |
| Köln | Herzzentrum der Universität Köln | Prof. Dr. med. Stephan Rosenkranz |
| Würzburg | Universitätsklinikum Würzburg, Deutsches Zentrum für Herzinsuffizienz | Prof. Dr. med. Stefan Störk |
| ***Qualified Site*** | | |
| Bad Friedrichshall | SLK-Kliniken Heilbronn GmbH–Klinikum am Plattenwald | Prof. Dr. med. Thomas Dengler |
| Bad Nauheim | Kerckhoff Klinik GmbH | Dr. med. Andreas Rieth |
| Bad Oeynhausen | Herz- und Diabeteszentrum NRW | PD Dr. med. Henrik Fox |
| Berlin | BG Unfallkrankenhaus Berlin | Dr. med. Sebastian Winkler |
| Berlin | Kardiologie im Spreebogen | PD Dr. med. Florian Krackhardt |
| Berlin | Universitätsmedizin Berlin, Charité–Benjamin Franklin | Prof. Dr. med. David M. Leistner |
| Berlin | Deutsches Herzzentrum Berlin | PD Dr. med. Felix Schönrath |
| Berlin | Sana Klinikum Lichtenberg | Prof. Dr. med. Fabian Knebel |
| Bielefeld | Klinikum Bielefeld gem. GmbH | Prof. Dr. med. Christoph Stellbrink |
| Bonn | Universitätsklinikum Bonn | PD Dr. med. Ulrich Becher |
| Coburg | Klinikum Coburg | Dr. med. Steffen Schnupp |
| Dortmund | St. Johannes-Hospital Dortmund | Prof. Dr. med. Helge Möllmann |
| Dresden | Praxisklinik Herz und Gefäße Dresden | Prof. Dr. med. Stefan Spitzer |
| Düsseldorf | Universitätsklinikum Düsseldorf | PD Dr. Ralf Westenfeld |
| Eisenach | St. Georg Klinikum Eisenach | Dr. med. Marcus Jahnecke |
| Erfurt | Helios Klinikum Erfurt GmbH | Prof Dr. med. Alexander Lauten |
| Essen | Universitätsklinikum Essen | PD Dr. med. Peter Lüdike |
| Halle (Saale) | Universitätsklinikum Halle (Saale) | Dr. med. Jörn Tongers |
| Hamburg | Universitäres Herz- und Gefäßzentrum Hamburg | PD Dr. med. Christina Magnussen |
| Hannover | Medizinische Hochschule Hannover | Prof. Dr. med. Tibor Kempf |
| Heidelberg | Universitätsklinikum Heidelberg | Prof. Dr. med. Lutz Frankenstein |
| Kaiserslautern | Westpfalz Klinikum GmbH | Prof. Dr. med. Burghard Schuhmacher |
| Karlsburg | Klinikum Karlsburg | Dr. med. Basil Alkhlout |
| Köln | St. Vinzenz-Hospital Köln | Dr. med. Stefan Winter |
| Leipzig | Leipzig Heart Institute GmbH | Prof. Dr. med. Gerd Hindricks |
| Ludwigsburg | Cardio Centrum Ludwigsburg-Bietigheim | PD Dr. med. Ralph Bosch |
| München | Deutsches Herzzentrum München | Prof. Dr. med. Christof Kolb |
| Neuruppin | Medizinische Hochschule Brandenburg | Prof. Dr. Michel Noutsias |
| Oldenburg | Klinikum Oldenburg | Dr. med. Lea Seidlmayer |
| Recklinghausen | Klinikum Vest | Prof. Dr. med. Frank Weidemann |
| Rostock | CardioConsil | Dr. med. Jens Placke |
| Trier | Krankenhaus Barmherzige Brüder | Neriman Osman |
| Villingen-Schwenningen | Schwarzwald-Baar Klinikum | Prof. Dr. med. Werner Jung |
| Winnenden | Rems-Murr Klinikum | Dr. med. Michael Sailer |
| ***Planned Site*** | | |
| Bad Berka | Zentralklinik Bad Berka | PD Dr. med. Torsten Schreiber |
| Berlin | Charité Universitätsmedizin Berlin | Prof. Dr. med. Friedrich Köhler |
| Bottrop | Knappschaftskrankenhaus Bottrop GmbH | Dr. med. Martin Christ |
| Münster | Universitätsklinikum Münster Zentralklinikum | Prof. Dr. med. Jürgen Sindermann |
| Dresden | Herzzentrum Dresden | Julia Fischer |
| Frankfurt | Kardiocentrum Frankfurt an der Klinik Rotes Kreuz | Ernst Geiß |
| Frankfurt | Klinikum Frankfurt Höchst | Prof. Dr. med. Hans Ulrich Hink |
| Heilbronn | SLK Kliniken Heilbronn GmbH | Prof. Dr. med. Marcus Hennersdorf |
| Leipzig | Universitätsklinikum Leipzig | Prof. Dr. med. Rolf Wachter |
| Ludwigshafen | Klinikum der Stadt Ludwigshafen a. Rh. gGmbH | Prof. Dr. med. Uwe Zeymer |
| Mainz | Universitätsmedizin Mainz | Prof. Dr. med. Philip Wenzel |

# Table S2. Time course of study procedures and patient contacts

|  | **Baseline** | | | **Post-Discharge** | | | |
| --- | --- | --- | --- | --- | --- | --- | --- |
| **Procedures** | **Inclusion** | **Implantation** | **Post-implantation** | **Telephone-**  **contact** | **3 months** | **6 months** | **12 months** |
| Patient consent | X |  |  |  |  |  |  |
| Screening (inclusion/ exclusion criteria) | X |  |  |  |  |  |  |
| Randomization | X |  |  |  |  |  |  |
| Demographics | X |  |  |  |  |  |  |
| General health, body weight, blood pressure, heart rate | X |  |  | X | X | X | X |
| Medical history and comorbidities | X |  |  |  |  |  |  |
| Cardiovascular medication | X |  | X |  | X | X | X |
| Guideline-directed medication | X |  | X | X | X | X | X |
| Other diagnostic^[[1]](#footnote-1)^ & therapeutic interventions |  |  |  |  | X | X | X |
| Laboratory parameters | X |  |  |  | X | X | X |
| Physical examination | X |  | X |  | X | X | X |
| Assessment NYHA functional class | X | X | X | X | X | X | X |
| Qualit of life (KCCQ, EQ-5D, PHQ-9, GAD-7) | X |  |  |  |  | X | X |
| Acceptance of monitoring |  |  |  |  |  | X | X |
| Procedural details of right heart catheterization and implantation (intervention group only) |  | X |  |  |  |  |  |
| Adverse events |  | X | X |  | X | X | X |
| Device- or system-related complications |  | X | X |  | X | X | X |
| PA pressure values and reports (intervention group only) |  |  | X | X | X | X | X |

1. **Further statistical analyses**

For the major secondary endpoint, changes in the KCCQ questionnaire will be compared between the intervention and control groups, where the treatment effect on KCCQ is the intervention group parameter based on a linear mixed model with repeated measurements, using the baseline KCCQ summary scores (overall summary score, OSS; total symptom score, TSS) as a covariate in the model. The model will include the change in the KCCQ summary scores from baseline to after 6 and 12 months, and will include fixed factors for intervention group, appointment and interaction of intervention groups per appointment. If no qualitative interaction is observed, the hypothesis will be tested using the main effect for the intervention group. The null hypothesis will be rejected if the two-tailed p-value is less than 5%. The analysis population will include the data up to the 12-month time point from subjects who have had a CardioMEMS™ HF sensor implanted and the data from subjects who only received basic care, and the hypothesis will be tested at the same time as the primary safety and efficacy endpoints. EQ-5D-5L scores will be analyzed in a comparable way. Mortality (HF-related, cardiovascular, non-cardiovascular and all-cause), unplanned hospitalizations (HF-related, cardiovascular, non-cardiovascular and all-cause), and adverse events will be evaluated using a Cox proportional hazard regression model with Anderson-Gill method for recurrent events. In addition, Cox proportional hazard models are implemented to analyze time to first events, including mortality and hospitalization. Hospitalization rates and mortality rates are estimated using the Kaplan-Meier method, and p-values are computed using the log-rank test. All reported analyses are performed using the intention-to-treat principle. All statistical tests will be 2‑sided with a significance level of 0.05.

1. **Trial organization**

**Project leader and coordinating investigator**

Stefan Störk, Universitätsklinikum Würzburg – Deutsches Zentrum für Herzinsuffizienz, Würzburg

**Steering Committee**

Stefan Störk (Chair), Christiane Angermann (Co-coordinating Investigator), Birgit Aßmus (Co-coordinating Investigator), Stephan Rosenkranz (Co-coordinating Investigator), Alexandra Bernhardt, Michael Böhm, Johannes Brachmann, Nikolaos Dagres, Stefan Frantz, Gerd Hindricks, Friedrich Köhler, Uwe Zeymer.

**Mentoring Sites**

Stefan Störk, Würzburg; Birgit Aßmus, Gießen; Stephan Rosenkranz, Cologne; Oliver Bruder, Essen.

**Biostatistics**

Thomas Riemer, IHF GmbH, Ludwigshafen.

**Funding**

The PASSPORT-HF trial is commissioned by the Federal Joint Committee (G-BA) in Germany for reimbursement and conditional coverage of health-care-related costs. As part of this program, an independent research grant for study costs has been provided by Abbott. Abbott has no role in the trial design, study management, data management and analysis or reporting of the findings.

**Data Safety Monitoring Board**

An independent data safety monitoring board (DSMB) has been established. It will review safety data on an ongoing basis during the trial in accordance with the DSMB charter. DSMB Members: Martin Cowie, Imperial College London, UK (Chair); Günter Breithardt, University Hospital Münster; Wilhelm Haverkamp, Charité Berlin; Jan G.P. Tjissen, University of Amsterdam, NL.

**Event Adjucation Committee**

Further, an independent endpoint adjudication committee (EAC) has been established, that will be blinded to study group assignment, and will review and adjudicate all deaths and hospitalizations using prospectively defined criteria in the EAC charter. The adjudicated data are used for outcomes regarding hospitalizations and deaths. EAC Members: Erland Erdmann, University Hospital Cologne (Chair), Ulrich Tebbe, Hospital Detmold; Markus Haass, Theresien-Krankenhaus Mannheim.

**Clinical Research Organization**

The PASSPORT-HF trial is structurally monitored by independent monitors from the CRO (IHF GmbH, Ludwigshafen) and the ZKSW (Zentrale für Klinische Studien, University Hospital Würzburg).

**Lead Ethics Committee**

The clinical trial has been approved by the appropriate medical ethics committee and review board (EC University Julius-Maximilians-Universität Würzburg; 236/19_mp-sc, 11-MAY-2020).

**Trial Registration**

The trial was registered under the number NCT04398654, clinical trial registration number) on 21 May 2020. Enrollment started in August 2020.

1. Includes: Computed tomography (CT), magnet resonance imaging (MRI), X-ray, coronary angiography. [↑](#footnote-ref-1)
